# The Clinical Rationale for the Sentry Bioconvertible Inferior Vena Cava Filter for the Prevention of Pulmonary Embolism

**DOI:** 10.1155/2019/5795148

**Published:** 2019-05-26

**Authors:** Michael D. Dake, Gary M. Ansel, Matthew S. Johnson, Robert Mendes, H. Bob Smouse

**Affiliations:** ^1^Departments of Medical Imaging and of Surgery, University of Arizona Health Sciences, Tucson, Arizona, USA; ^2^Department of Cardiology & Vascular Medicine, OhioHealth/Riverside Methodist Hospital, Columbus, Ohio, USA; ^3^Department of Vascular & Interventional Radiology, Indiana University School of Medicine, Indianapolis, Indiana, USA; ^4^Department of Vascular Surgery, UNC Rex Hospital, NC Heart and Vascular Research, Raleigh, North Carolina, USA; ^5^Department of Interventional Radiology, OSF Saint Francis Medical Center, Peoria, Illinois, USA

## Abstract

The Sentry inferior vena cava (IVC) filter is designed to provide temporary protection against pulmonary embolism (PE) during transient high-risk periods and then to bioconvert after 60 days after implantation. At the time of bioconversion, the device's nitinol arms retract from the filtering position into the caval wall. Subsequently, the stable stent-like nitinol frame is endothelialized. The Sentry bioconvertible IVC filter has been evaluated in a multicenter investigational-device-exemption pivotal trial (NCT01975090) of 129 patients with documented deep vein thrombosis (DVT) or PE, or at temporary risk of developing DVT or PE, and with contraindications to anticoagulation. Successful filter conversion was observed in 95.7% of patients at 6 months (110/115) and 96.4% at 12 months (106/110). Through 12 months, there were no cases of symptomatic PE. The rationale for development of the Sentry bioconvertible device includes the following considerations: (1) the period of highest risk of PE for the vast majority of patients occurs within the first 60 days after an index event, with most of the PEs occurring in the first 30 days; (2) the design of retrievable IVC filters to support their removal after a transitory high-PE-risk period has, in practice, been associated with insecure filter dynamics and time-dependent complications including tilting, fracture, embolization, migration, and IVC perforation; (3) most retrievable IVC filters are placed for temporary protection, but for a variety of reasons they are not removed in any more than half of implanted patients, and when removal is attempted, the procedure is not always successful even with advanced techniques; and (4) analysis of Medicare hospital data suggests that payment for the retrieval procedure does not routinely compensate for expense. The Sentry device is not intended for removal after bioconversion. In initial clinical use, complications have been limited. Long-term results for the Sentry bioconvertible IVC filter are anticipated soon.

## 1. Introduction

Treatment with anticoagulant agents is the established primary treatment for venous thromboembolic (VTE) disease. For many patients, however, anticoagulation is contraindicated or not effective, or it has to be discontinued during periods of high risk for pulmonary embolism (PE). In these situations, inferior vena cava (IVC) filters are recommended in accordance with careful selection criteria developed by various societies and expert panels [1, 2]. The purpose of IVC filter placement is temporary or permanent mechanical protection against PE, which is a major cause of morbidity and mortality. Several systematic reviews of the literature have shown that IVC filters are effective at providing such mechanical protection [3–5].

Permanent IVC filters became available beginning in the 1970s [6]. In response to adverse events (in particular IVC thrombosis) and to concerns about long-term durability and safety, retrievable IVC filters were developed in the early 2000s [6]. More than a dozen retrievable IVC filters have become commercially available in the United States [7]. Retrievable IVC filters were approved by the US Food and Drug Administration (FDA) for permanent as well as transient uses, but there was an expectation that the majority of the filters would be retrieved, thus providing the benefit of short-term mechanical caval filtration while avoiding complications associated with permanent placement. Accordingly, with the design priority of facilitating retrieval by limiting endothelialization, their caval mounting can be relatively less secure than that of permanent filters [6, 8]. The long-term safety and efficacy of retrievable IVC filters as a class have not been established [2].

While retrievable IVC filters have been designed with awareness that — for good clinical reason or due to patients being lost to follow-up — some will not be removed, nevertheless in practice an unanticipated proportion of these devices have been allowed to remain in place after the need for temporary PE prophylaxis has passed. Less than half of retrievable IVC filers are removed, and, in some studies, retrieval rates have been as low as 8.5% [3, 9, 10]. A number of studies and case reports have been published describing time-dependent complications — such as tilting, fracture, embolization, migration, or IVC perforation — associated with retrievable IVC filters that remain indwelling [7]. Beginning in 2010, the FDA has issued safety communications about retrievable IVC filters [11]. In 2016, systematic follow-up of patients with retrievable IVC filters at 3 months became a Medicare quality metric [12].

**Figure 1:**
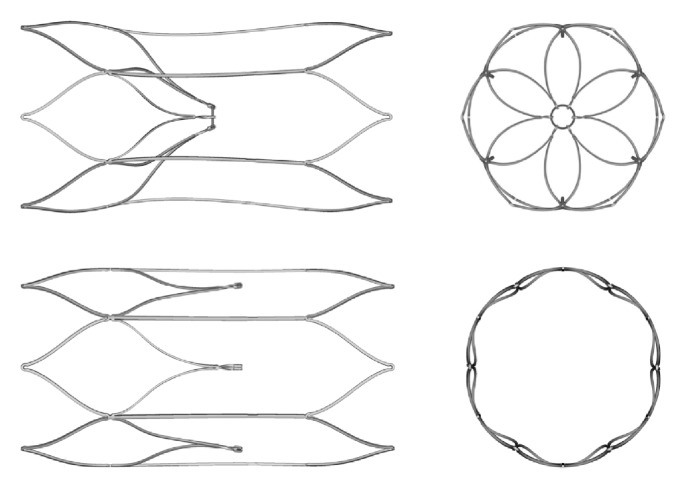
The Sentry bioconvertible IVC filter in filtering (above) and bioconverted (below) configurations, with corresponding axial views on the right.

By requiring a follow-up procedure for device removal, retrievable IVC filters were designed to offset the risk of adverse events associated with permanent mechanical caval filtration such as late IVC thrombosis and filter migration. To achieve that same end but avoid the necessity of a secondary removal procedure, the Sentry IVC filter (BTG Vascular, Bothell, WA) was designed to provide protection during the high-PE-risk period (up to 60 days) and then bioconvert from a filtering to a nonfiltering configuration (Figure 1). The means of the conversion for the Sentry device is hydrolytic biodegradation of a filament that holds together the six pairs of arms that form the filter cone in the central portion of the caval lumen. This filament is composed of poly-*p*-dioxanone (PPDO), a hydrolyzing synthetic polymer that has been widely used in biodegradable devices such as sutures [13]. After the PPDO filament biodegrades, the arms are released and retract to the caval wall to become endothelialized along with the stable stent-like device frame, reducing the risk of filter migration or embolization and maintaining vessel patency. The Sentry's bioconversion mechanism thus obviates the need for a separate secondary removal procedure as with retrievable filters.

In this article, we discuss the clinical rationale for the development of the Sentry bioconvertible IVC filter. We discuss considerations that informed the Sentry development: (1) evidence that the period of highest risk of PE for the vast majority of patients occurs within the first 60 days after an index event, with most of the PEs occurring in the first 30 days; (2) evidence that retrievable IVC filters, which have been a focus of safety communications from the FDA [11, 14], are associated with time-dependent complications, many arising due to the design features of the devices and because they are not routinely removed as intended; (3) evidence that when removal of retrievable IVC filters is actually attempted, the procedures are frequently complex — with a failure rate of approximately 10% [9] — requiring advanced techniques such as the use of forceps, lead extraction devices, or lasers; and (4) evidence — based on an analysis of Medicare hospital data on cases of IVC filter removal — suggesting that the need for complex filter retrievals presents an economic burden, with reimbursement for the procedure not routinely compensating for expense. We conclude the article by describing the clinical development program of the Sentry bioconvertible IVC filter and studies of the device to date.

## 2. Clinical Rationale for the Sentry Bioconvertible IVC Filter

### 2.1. The Timing of PE after an Index Event

Risk factors for PE include a history of deep vein thrombosis (DVT); recent surgical procedures; hospitalization for cancer and chronic conditions; prolonged inactivity or immobility; traumatic injury; obesity; and advanced age [15]. Contemporary data from large-population studies support the premise that the period of highest risk for PE in patients with temporary contraindications to anticoagulants occurs early, with the clear majority of PEs diagnosed within 30 days of an index event (hospitalization, traumatic injury, surgery).  Table 1 summarizes the findings regarding the incidence and time course of PE in trauma patients, and Table 2 summarizes data on the postoperative incidence and time course of PE in surgical patients.

**Figure 2:**
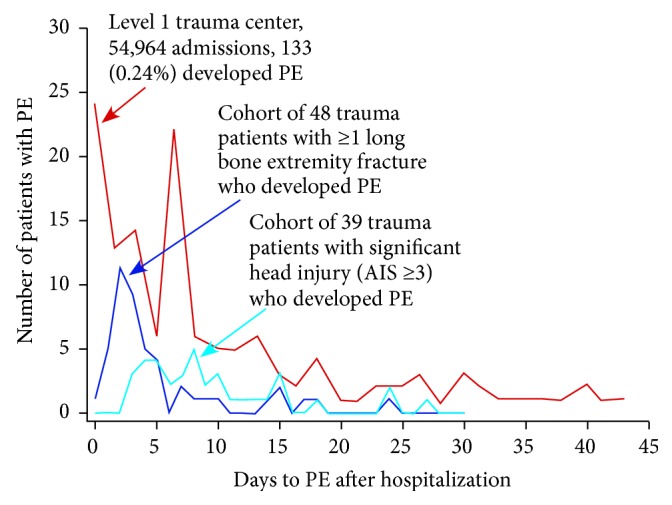
Time course to pulmonary embolism (PE) in patients at level 1 trauma centers. AIS = Abbreviated Injury Scale. Adapted from Brakenridge et al. [19] and Coleman et al. [17]

In a retrospective chart review of 25,658 trauma patients during the years 2001 to 2004 at four level 1 trauma centers, Sing et al. [16] found an 0.6% incidence of PE, with a mean time from injury to PE diagnosis of 7.9 ± 8.1 days and with the latest event occurring at 43 days. In another large retrospective chart review, by Coleman et al. [17], involving data for 54,964 patients at three level 1 trauma centers from 2007 to 2013, the incidence of PE was 0.24%, with a median time to PE diagnosis of 4 hospital days (interquartile range [IQR] 1 to 8 days).  Figure 2 diagrams the time course for the 133 PE diagnoses through 43 days in that study, along with the timing of PE through 30 days in 48 patients with ≥1 long bone fracture and 39 with significant head injury between 2003 and 2007 in level 1 trauma center data reported by Brakenridge et al. [19] Of the 108 PE diagnoses, overall in the study by Brakenridge et al., 25% were within the first 72 hours, 50% within the first 4 days, and 95% within 20 days [19]. In a retrospective chart review by Batty et al. [20], the mean time to PE for trauma patients was 12 days after injury, with a range extending to 48 days.

**Figure 3:**
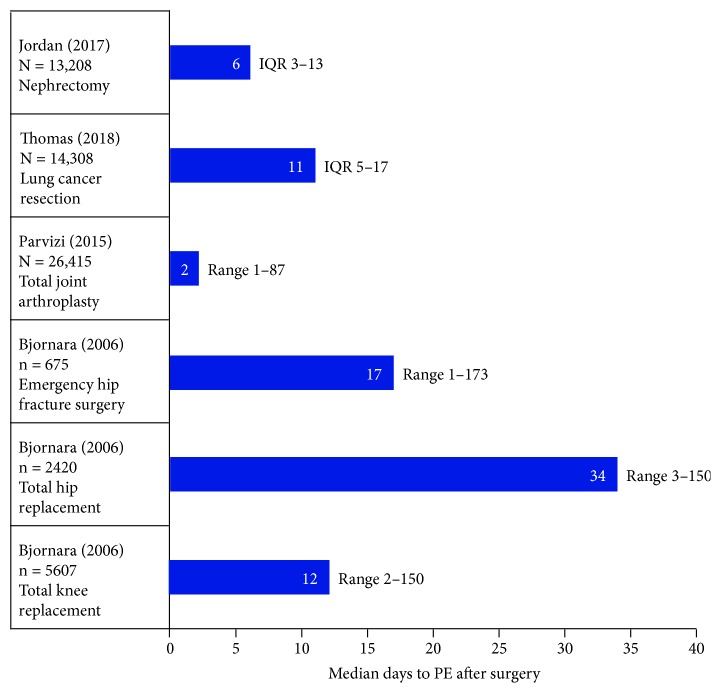
Median days to pulmonary embolism (PE) in large surgery populations. IQR = Interquartile range. Data from Jordan et al. [25], Thomas et al. [26], Parvizi et al. [31], and Bjornara et al. [29].

In a study of 5607 patients who underwent major joint surgery at a Norwegian hospital between 1989 and 2001, Bjornara et al. [29] found that the median time to PE diagnosis was 17 days (range 1 to 173 days) following emergency hip fracture surgery, 34 days (range 2 to 150 days) following total hip replacement, and 12 days (range 2 to 150 days) following total knee replacement (Figure 3). In a study of 115 PE patients in a population of 111,773 who underwent general, orthopedic, and other surgical procedures from 1999 to 2004 at a North Carolina medical center, Hope et al. [22] found that the mean time to PE diagnosis was 2.6 days for patients <40 years of age, 11.2 days for those 40 to 60 years of age, and 6.7 days for those >60 years. Of 1602 patients with VTE following a variety of surgical procedures in the international RIETE registry, as reported by Arcelus et al. [23], 787 were diagnosed with PE — 19% during the first 7 days, 48% during the first 15 days, and 77% during the first 30 days. Three recent reports, based on the American College of Surgeons National Surgical Quality Improvement Program database, found a mean time to postoperative PE for thoracolumbar surgery patients of 9.4 ± 7.3 days [24], a median time to PE for nephrectomy patients of 6 days (IQR 3 to 13 days) [25], and a median time to PE for lung cancer resection patients of 11 days (IQR 5 to 17 days) [26]. Recently among numerous studies of postoperative PE in orthopedic surgery patients, Parvizi et al. [31] found an incidence following total joint arthroplasty of 1.07% (283/26,415), with a median time to diagnosis of 2 days (range 1 to 87 days) — 81% within the first 3 days, 89% within the first week, and 94% within 2 weeks. In adult femur fracture patients who underwent computed tomography (CT) within 72 hours after admission at a Korean medical center, Kim et al. [32] found a PE incidence of 2.2% (28/453) — with the PE detected in 57.1% in the first 24 hours after injury and in 89.3% in the first 48 hours.

### 2.2. Time-Dependent Complications of Retrievable IVC Filters

Clinician and public concern about high rates of complications associated with retrievable IVC filters followed numerous reports in the literature and was amplified by the issuing of a safety communication from the FDA in April 2010 [11]. The safety communication has been associated with a decrease in the utilization of IVC filters [33]. Several systematic reviews of retrievable IVC filters have assessed overall and specific device-related complications.  Table 3 shows reported rates for key filter-related complications from three systematic reviews [3, 7, 9] and from two analyses of adverse events that were voluntarily self-reported by clinicians to the FDA Manufacturer and User Facility Device Experience (MAUDE) database [3, 34].

The April 2010 FDA safety communication stated that, because of the number of reports of device-related adverse events received since 2005, the FDA was concerned that retrievable IVC filters, “intended for short-term placement, are not always removed once a patient's risk of PE has subsided,” and that retrieval is important because “long-term risks associated with IVC filters include but are not limited to DVT, filter fracture, filter migration, filter embolization, and IVC perforation” [11]. In May 2014, the FDA updated the April 2010 safety communication, again urging physicians to consider removing retrievable IVC filters as soon as PE protection is no longer needed [14].

In the 2014 update, two FDA initiatives were highlighted. The first was development of a quantitative decision analysis, using data from the literature about IVC filters, to assess whether a time point could be identified beyond which the risk of keeping an IVC filter in place might outweigh the benefits. This mathematical model, published by Morales et al. in 2013 [35], suggested that if a patient's transient risk requiring mechanical protection against PE has passed, the risk/benefit profile favors removal of the IVC filter between 29 and 54 days after implantation. This analysis and supporting data suggested that the vast majority of cases of PE occur well within 60 days of an index event, affirming the selection of a 60-day biodegradation window for the filament holding the filter-cone-forming arms of the Sentry IVC filter.

The second FDA initiative involved collection of data for IVC devices currently marketed in the United States in order to reassess their effectiveness and safety. Two options were given to manufacturers for providing data: (1) postmarket surveillance or (2) participation in the Predicting the Safety and Effectiveness of Inferior Vena Cava Filters (PRESERVE) trial (NCT02381509). Sponsored by the IVC Filter Study Group Foundation, a joint collaboration between the Society of Interventional Radiology (SIR) and the Society for Vascular Surgery, PRESERVE is a multicenter, prospective, open-label, nonrandomized registry of commercially available IVC filters from 6 manufacturers placed in patients for the prevention of PE. Enrollment was begun in October 2015. As of August 2018, according to the SIR Foundation, 4 of the 6 originally included filters had reached their 300-patient cap and had their enrollment closed. Data are expected from PRESERVE in 2019.

In 2014, Andreoli et al. [34] compared retrievable and permanent IVC filters in terms of adverse events reported to MAUDE for the period of January 2009 through 2012. For statistical purposes, the investigators estimated that 75% of the filters present in the population during that time period were retrievable IVC filters. Of the 1696 MAUDE adverse events associated with 1057 IVC filters for that time period, 86.8% involved retrievable IVC filters, whereas only 13.2% involved permanent IVC filters (p <0.0001). For every specific complication — including tilting, fracture, embolization, migration, IVC perforation, and venous thromboembolism — absolute numbers were significantly higher for retrievable IVC filters than for permanent IVC filters.

In a 2011 systematic review of retrievable IVC filters, Angel et al. [3] included an analysis of MAUDE data on retrievable IVC filters, adding the insight that almost all MAUDE events (93%) occurred after 30 days. The systematic review identified 37 studies in the literature in which 6834 patients received retrievable IVC filters. The mean follow-up of the studies was 9.9 months (range 2 to 25 months). The rate of device-related complications associated with retrievable IVC filters was low, the investigators stated, but time-dependent, with few complications reported within 30 days of filter implantation. The mean retrieval rate was “extremely low” 34% (range 12% to 45%).

In a 2018 systematic review of retrievable IVC filters conducted by Jia et al. [9], the retrieval rate was found to be similarly low at 34.9%, although 85% of the IVC filter placements were intended to be temporary. This systematic review identified 103 studies in which 20,319 patients received retrievable IVC filters. The investigators noted a low incidence of complications but underscored that median follow-up was only 140 days (range 18 to 3279 days) and that complications increased with longer indwelling times. Retrieval attempts failed for 13.7% of all implanted IVC filters.

In 2016, Deso et al. [7] analyzed complications according to permanent or retrievable IVC filter brands and types. Many different IVC filters and filter design types have been approved by the FDA since 1980. The investigators identified 9 permanent IVC filter brands, of which 3 were no longer manufactured in 2016, and they identified 14 retrievable IVC filter brands, of which 7 were no longer manufactured in 2016 [7]. The Deso et al. systematic review found 73 articles reporting IVC filter complications. For the retrievable IVC filters identified in this analysis, the proportions with reported rates >10% in selected complication categories are included in Table 3. In this review — as in the Andreoli et al. review of the MAUDE database [34] — the most commonly reported complications varied among the brands of retrievable filters. The rate of filter fracture for the Bard Recovery IVC retrievable filter (C.R. Bard Peripheral Vascular, Tempe, AZ) was reported to be as high as 39.5%; for the Cordis OptEase/TrapEase IVC filter (Miami Lakes, FL), the filter-fracture rate was reported to be as high as 50%. Rates of filter penetration for the Bard Recovery and the Cook Celect (Bloomington, IN) retrievable IVC filters were reported to exceed 90% [7].

Among the IVC filter brands included in the Deso et al. analysis, the most common design type, used in more than half the devices, was conical. When such a design is involved, the investigators advised, routine assessment for IVC filter perforation should be performed. With such devices, the endothelialization-limiting (and intended retrieval-facilitating) point contact on the part of the filter legs can lead to penetration of the caval wall and to instability and tilting. For device designs with cylindrical frame elements, the investigators advised assessment for IVC occlusion [7]. In the case of the OptEase retrievable device with filter elements at each end of a box-like cylindrical frame, the high metal content within the lumen and associated abnormal flow characteristics can induce occlusion [36].

Other studies have specifically examined complications with retrievable IVC filters, sometimes focusing on institutional experiences [10, 37], specific IVC filter brands [38–40], or difficulties that complications present in relation to attempts at IVC filter retrieval [41–44]. A notable analysis was another systematic review by Jia et al. [45] that examined the effects of caval penetration by IVC filters. A total of 88 clinical studies and 112 case reports qualified for analysis, representing 9002 patients and 15 filter types. The overall penetration rate was 19% of patients, and almost one in five of those penetrations showed evidence of organ/structure involvement. Among patients with penetration, 8% were symptomatic, 45% were asymptomatic, and 47% had unknown symptomatology. The most frequently reported symptom was pain (77%), and major complications were reported in 5% of patients, including 2 deaths. The investigators stated that caval penetration by IVC filters occurs frequently but is poorly recognized, although almost one tenth of the penetrations caused symptoms.

In summary, the recognition that prompted the development of retrievable IVC filters — that a device providing caval mechanical filtration is increasingly likely to develop complications over time — has also held true for retrievable IVC filters, which were specifically designed with modification to the caval attachment sites so that they could be removed. To date, there have been no randomized controlled trials directly comparing permanent and retrievable IVC filters. Review of the MAUDE database, for example, suggests that, after the need for mechanical PE prophylaxis has passed, complication rates might indeed be higher with retrievable IVC filters than with permanent IVC filters [34].

### 2.3. The Complexity of IVC Filter Retrieval

The 2010 FDA safety communication stimulated efforts to improve upon the low retrieval rates of IVC filters. Because lack of systematic follow-up of patients receiving IVC filters was identified as an important reason for the absence of attempts at retrieval, several dedicated tracking programs were set up to improve filter retrieval rates. After a filter registry was initiated in one program, retrieval rates improved from 15.5% to 31.5% [46]. Another program, which sent automated letters to physicians and made automated follow-up appointments, improved retrieval rates from 8% to 52% [47]. A third program, which focused on both physician education and patient tracking, improved retrieval rates from 38.9% to 54%, while use of IVC filters decreased by 18.7% [48]. Since 2012, in the Medicare population, when a unique Common Procedure Terminology (CPT) code (37193) was introduced for IVC filter retrieval, the net annual filter retrieval rate increased from 6.9% in 2012 to 22.1% in 2016 [49].

The 2010 FDA safety communication raised clinician awareness that retrievable IVC filters can in fact be challenging to remove. Significant filter tilt, an embedded filter, a caval strut perforation, a filter fracture, or the presence of thrombus within the filter — in addition to ordinary tissue ingrowth and strut epithelialization — can make retrieval problematic [50, 51]. Dinglassan et al. [52] examined preretrieval filter characteristics noted by CT that were associated with complicated retrieval procedures, comparing 48 patients with complicated retrievals versus 48 control patients with uncomplicated retrievals. They found that mediolateral and anteroposterior tilt angle, degree of perforation, and dwell time were higher for the complicated retrieval group than for the uncomplicated retrieval group (p <0.01). The odds of complicated retrieval were increased by an embedded tip appearance on CT (odds ratio [OR] 129, p <0.0001); a tilt angle >15 degrees (OR 33, p <0.0001), a higher-grade perforation (OR 10.7, p <0.001), and dwell time >180 days (OR 2.3, p <0.05).

Recent reviews have analyzed standard and advanced techniques for achieving IVC filter retrieval, including such methods as laser thermal and endobronchial forceps dissection [50, 53, 54]. Case reports have described open surgical removal of IVC filters, a last resort for patients with symptoms referable to filter strut penetration, such as chronic back and abdominal pain, after failed endovascular attempts [55]. In a retrospective analysis of filter retrieval over a 10-year period in 217 patients, Al-Hakim et al. [56] found that the routine standard technique of snaring the filter hook and sheathing the filter was successful in 73.2% of patients, failing most frequently because of significant filter tilt or an embedded hook. For those patients for whom the routine technique failed, advanced techniques had a success rate of 94.7% but required significantly longer fluoroscopy time and had a significantly higher complication rate (5.3% vs. 0.4%, p <0.05).

### 2.4. The Cost of IVC Filter Retrieval

In order to analyze Medicare claims associated with IVC filter retrieval, we extracted cost and payment data from the 2016 Medicare outpatient hospital standard analytical file for CPT code 37193, the code specific for IVC filter removal [57]. A total of 6687 outpatient IVC filter retrieval procedures were billed to Medicare in 2016; of these procedures, 1140 (17.0%) required the use of anesthesia. Medicare payments were calculated from final claims for which Medicare was the primary payer, payment was not denied, and there were no other outlier payments. Costs were estimated by applying the 2016 cost-to-charge ratio for each hospital, as determined by the US Center for Medicare and Medicaid Services, to the charges billed by each hospital for CPT code 37193. As seen in Table 4, in 2016, Medicare payment for outpatient IVC filter removal did not routinely appear to cover hospital costs. The median payment was $1767.49 (interquartile range $1683.78–$1921.01), but the median cost was $3129.57 (interquartile range $2457.04–$4167.18). Consequently, median Medicare payment was only 56% of median cost in 2016 for removal of IVC filters, a difference of $1362.08, and the median interquartile range for payment was narrower than the median interquartile range for cost. No doubt, part of the shortfall in 2016 Medicare payments for the costs of IVC filter removal was covered by copayments from Medicare patients, but such copayments do not generally exceed 20% of the Medicare payment. The Sentry IVC filter, again, is not designed to be retrieved after bioconversion and therefore obviates the need for the retrieval procedure.

## 3. Sentry IVC Filter Description and Performance

**Figure 4:**
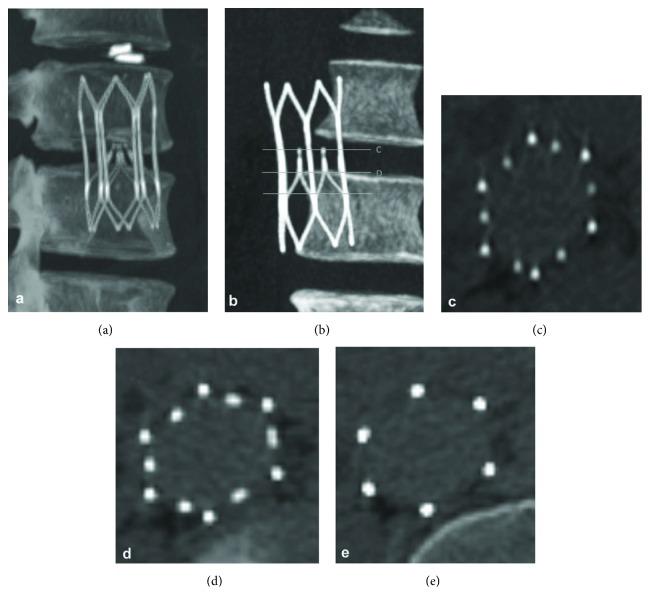
Computed tomography imaging of the Sentry bioconvertible IVC filter for a single patient. (a) Coronal image acquired as part of helical dataset 1 month after filter placement, showing the Sentry device in filtering configuration. (b) Coronal image at 6-month follow-up showing the device in bioconverted configuration. ((c), (d), (e)) Axial views of the bioconverted device at 6-month follow-up, with the images keyed to the coronal view in (b). Reprinted with permission from Dake et al. [60]

The Sentry IVC filter is made from a single piece of laser-cut nitinol, which is formed into a stable self-centering cylindrical frame with an integral filter cone consisting of 6 pairs of arms held together in the center of the IVC lumen by means of the bioabsorbable PPDO filament. The cylindrical nitinol frame is designed to expand upon deployment to appose the caval wall (without point contact), such that it concentrically and longitudinally optimizes flow and distributes radial force in order to decrease device tilting, migration, perforation, and fracture as endothelialization and neointimal healing commence. Six fixation barbs (4 in the cranial direction and 2 in the caudal direction) are located on the nitinol frame to keep the device securely in place and minimize device migration. When the bioabsorbable filament hydrolyzes and releases the filter-cone-forming arms after 60 days, they retract to the caval wall by means of the nitinol shape memory to their nonfiltering set position for endothelialization along with the stable stent-like device frame (Figure 4). This design allows temporary protection against PE followed by maintenance of IVC lumen patency.

The Sentry device is indicated for use in IVC with diameters between 16 and 28 mm and has a maximum deployed length of 57.7 mm. The filter comes preloaded in a loading tool, which is attached to a custom introducer sheath for deployment. It is suitable for a femoral or jugular approach. The filter is advanced through the introducer sheath using a pusher, which is supplied with the device. Once the device is in the intended location, the pusher is held stationary and the introducer sheath is retracted to execute deployment.

### 3.1. Animal Studies

The performance and stages of incorporation of the Sentry IVC filter were examined in an ovine model. Sheep were used, as required by the FDA, because their IVC sizes are closer to IVC sizes in humans than are IVC sizes in pigs or dogs [58]. A total of 24 Sentry study devices and 1 control retrievable IVC filter were implanted in the infrarenal IVC of 25 sheep, with daily monitoring and imaging and posttermination necropsy. The devices and animals were treated and evaluated in two distinct cohorts: (1) early-incorporation analysis cohort, with termination and necropsy performed ≤98 days after implantation (n = 10 study devices) and (2) late-incorporation analysis cohort, for assessment of device incorporation (cylindrical frame and retracted filter arms), with termination and necropsy planned for 180 ± 30 days after implantation (n = 14 study devices and 1 control device).

**Figure 5:**
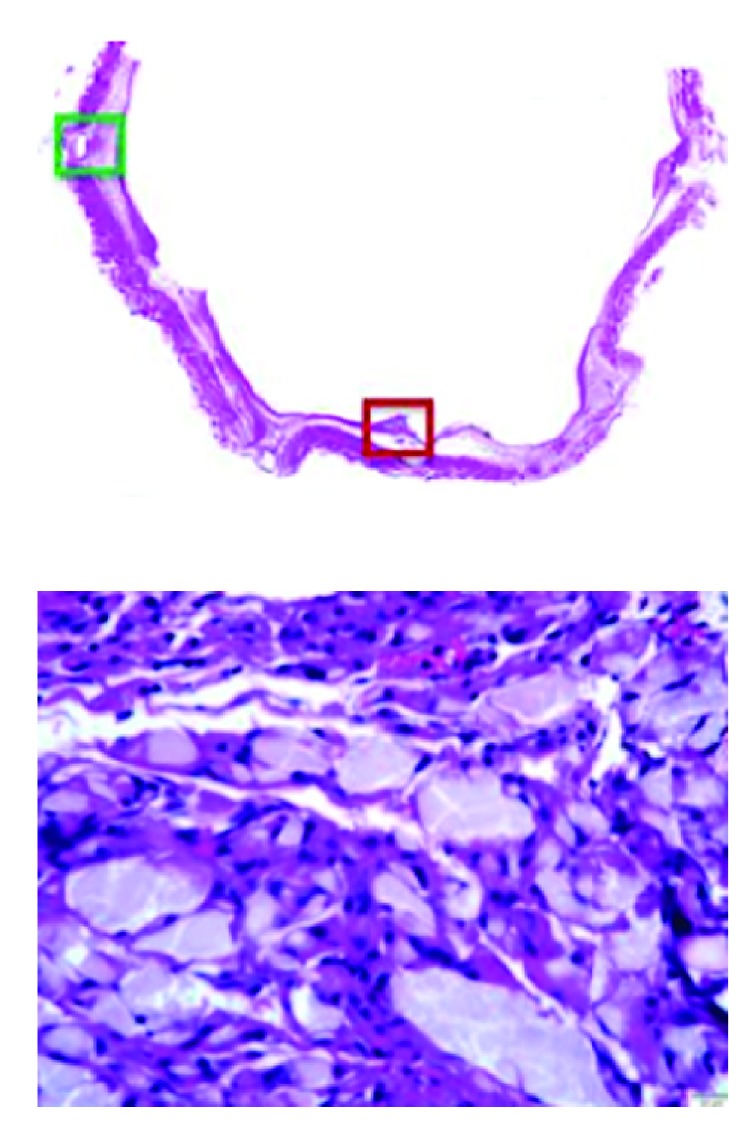
Histological images of the integration of the Sentry filter arms into the wall of the inferior vena cava. Above: section cut through the tips of the filter arms stained with hematoxylin and eosin. Below (dark red box in image above): residual filament material surrounding chronic inflammatory cells adjacent to the fully incorporated tip of a filter arm. Reprinted with permission from Gaines et al. [59]

The results of the animal studies have been reported by Gaines et al. [59]. In all 24 animals that received study devices, deployment and positioning in the filtering configuration in the center of the IVC lumen were successful, and pre- and posttermination examinations confirmed that all filters had bioconverted as intended, leaving the IVC lumens patent. For the animals terminated at ≤98 days, imaging and necropsy showed that the stabilizing stent part of the Sentry frame was incorporated in the vessel wall, and the filter arms were retracted. For the animals terminated at 180 ± 30 days, imaging, necropsy, and histopathology confirmed that the filter arms as well as frames were stably incorporated in the vessel wall and that the IVC lumens were unobstructed and patent.  Figure 5 shows a representative section from the histological analysis through the tips of the Sentry filter arms, stained with hematoxylin and eosin, confirming the integration of the filter arms into the wall of the IVC. The high-power magnification of the section shows the residual PPDO filter element surrounded by chronic inflammatory cells adjacent to a fully incorporated tip of a filter arm. Through 180 days, there were no filter-related complications.

### 3.2. Pivotal SENTRY Clinical Trial

The prospective, multicenter, nonrandomized, single-arm SENTRY Clinical Trial (NCT01975090) was conducted at 23 sites in the United States (n = 20), Belgium (n = 2), and Chile (n = 1). Patients eligible for inclusion were over 18 years of age and were determined by their physicians to be at temporary (<60 days) risk of PE. All patients had documented DVT or PE or were at high risk of developing DVT or PE and had a contraindication to or failure of anticoagulation. These indications for enrollment were consistent with American College of Radiology (ACR) and SIR practice and quality improvement guidelines [2, 61]. Patients were monitored by radiography, CT, and CT venography in order to assess filtering configuration through 60 days, filter bioconversion, and incidence of PE and filter-related complications through 12 months.

The one-year results of the SENTRY Clinical Trial have been reported by Dake et al. [60]. In summary, 129 subjects were enrolled, 87 with a therapeutic indication and 42 with a prophylactic indication. Clinical success — defined as successful filter deployment, freedom from new symptomatic PE through 60 days before filter bioconversion, and 6-month freedom from filter-related complications — was achieved in 111 of 114 evaluable patients (97.4%, 95% CI 92.5%-99.1%), and device deployment was successful in all patients. The rate of freedom from new symptomatic PE was 100% through 60 days (n = 129, 95% CI 97.1%-100.0%) and 100% (n = 111, 95% CI 97.1%-100.0%) through 12 months. Through 60 days, the rate of new or worsening DVT was 7.8% (none confirmed to be device related), similar to recent findings for retrievable filters. Filter bioconversion was successful for 95.7% (110/115) at 6 months and 96.4% (106/110) at 12 months. Two patients (1.6%) developed symptomatic caval thrombosis during the first month; neither experienced recurrence after successful treatment with thrombectomy and thrombolysis. There was no filter tilting, migration, embolization, fracture, or caval perforation, and no filter-related death through 12 months. Overall, through 1 year, the Sentry IVC filter provided safe and effective mechanical protection against PE.

The reasons for the nonconversion of the Sentry IVC filter in 4 patients as of 12-month follow-up cannot be accurately determined. In preclinical studies, the presence of fibrin strands was occasionally noted at the ends of filter arm, and such a circumstance could delay or prevent separation of the arms [59]. In context, however, the 96.4% rate of bioconversion far surpasses reported retrieval rates for IVC filters [3, 9, 10]. In a relatively small percentage of patients, then, the risks associated with nonconversion may be seen as similar to the risks associated with a permanent or unretrieved IVC filter. In the 12-month follow-up of the SENTRY Clinical Trial, no patient experienced clinical sequelae related to device nonconversion, and in particular no PE or caval thrombosis.

Regarding the situation in which the Sentry IVC filter has captured a large thrombus prior to bioconversion, the protocol-mandated 1-month CT venography in the SENTRY Clinical Trial revealed the presence of thrombus in 15.8% of patients (18/114), and this thrombus was symptomatic in 2 patients. None of these patients experienced a PE through 12 months. At 2 months, data from a CT venogram was available for 10 of the 18 patients: in 3, the clot had completely resolved; in 5, the size of the clot was reduced; and in one, the clot was slightly longer (increase of <4 mm) while its width was decreased. In one patient imaging was not of sufficient quality to judge any change in size, although the continuing presence of thrombus was confirmed. The Sentry IVC filter was designed not to tilt, and any trapped thrombus is thus retained in the center of the vessel where the body's own lysis capabilities may be maximized, reducing the size of the trapped clot to subclinical levels. It is unlikely that the trapped clot is “free floating,” as it tends to become adherent to the filter elements.

Longer-term follow-up of the SENTRY Clinical Trial is underway, and 24-month follow-up will include CT venography, further reporting of complications, and confirmation of IVC patency.

## 4. Summary

Risk factors for PE include a history of deep vein thrombosis (DVT); recent surgical procedures; hospitalization for cancer and chronic conditions; prolonged inactivity or immobility; traumatic injury; obesity; and advanced age [15]. The Sentry bioconvertible inferior vena cava (IVC) filter provides temporary protection against PE in patients during such high-risk periods. When the device's central filament hydrolyzes after a protection period of 60 days, the filtering arms are designed to retract into the caval wall by means of nitinol shape memory and the stable stent-like device frame is endothelialized, maintaining lumen patency. Complications with the Sentry are limited, and, as the device is not intended for removal, no secondary procedure is required. The Sentry bioconvertible IVC filter has been evaluated in a multicenter investigational-device-exemption pivotal trial of 129 patients, with successful filter conversion occurring for 95.7% of patients at 6 months (110/115) and 96.4% of patients at 12 months (106/110). Through 12 months in the trial follow-up, there have been no cases of PE.

Considerations that figured in the clinical rationale for development of the Sentry device included (1) contemporary data on PE timing from large-population studies supporting the premise that the period of highest risk for PE in patients with temporary contraindications to anticoagulants occurs early, with the clear majority of PEs diagnosed within 30 days of an index event (hospitalization, traumatic injury, surgery), well within the Sentry's targeted 60-day window for automatic device conversion; (2) evidence that retrievable IVC filters are associated with time-dependent complications, many arising due to the design features of the devices and because they are not routinely removed as intended; (3) evidence that when removal of retrievable IVC filters is actually attempted, the procedures are frequently complex and require advanced techniques; and (4) evidence — based on an analysis of Medicare hospital data — suggesting that the need for complex filter retrievals presents an economic burden, with reimbursement for the procedure not routinely compensating for expense.

## Figures and Tables

**Table 1:** Postinjury incidence and time course of pulmonary embolism in trauma patients.

Author (year)	Trauma population	PE incidence n/N (%)	Postinjury time course of PE	Comments
Owings [18] (1997)	Patients admitted to single level 1 university trauma center from 1990 to 1995	63/18,255 (0.3%)	PEs occurred from days 1 to 30 following traumatic injury; one quarter of PEs occurred within first 4 days	58 (92%) of the PE patients had at least one of the accepted risk factors for VTE

Sing [16] (2006)	Retrospective chart review of trauma patients with PE 2001 to 2004 at four level 1 trauma centers	146/25,658 (0.6%)	Mean time to PE 7.9 ± 8.1 days, range 1 to 43 days; 24 PE occurred ≥15 days after injury, 16 (11%) ≥21 days (including 1 fatal)	IVC filters not deployed in these patients; the authors argued that the duration of filter implantation should be individualized

Brakenridge [19] (2011)	Patients admitted to single level 1 urban trauma center from 2003 to 2007	108/17,736 (0.6%)	25% within first 72 hours, 50% within first 4 days, 95% within 20 days; 6 cases diagnosed between 24 and 74 days	Long bone fractures independently predicted early PE (≤4 days, OR 2.8); severe head injuries associated with late PE (>4 days, OR 11.1)

Batty [20] (2012)	Retrospective chart review of data collected prospectively July 2001 to July 2008 in Australian hospital trauma registry	45/6344 (0.71%)	Mean time to PE 12 days postinjury (range 0 to 48 days)	Independent predictors of PE: absence of IVC filter, number of injuries to lower limb, central venous catheterization

Coleman [17] (2015)	Retrospective chart review of trauma patients with PE 2007 to 2013 at three level 1 trauma centers	133/54,964 (0.24%)	Median time from admission to diagnosis of PE 4 hospital days (IQR 1–8), when 79 of PE occurred; 42.9% of PE diagnosed within first 3 days (early PE), 57.1% with late PE	Long bone fracture with extremity AIS score ≥3 significantly predicted early PE (p <0.05); severe brain injury, spinal cord injury, and blood transfusion ≤24 hours predicted late PE

Van Gent [21] (2017)	Retrospective chart review of all adult patients who had ≥1 duplex ultrasound July 2006 through December 2011 at San Diego level 1 trauma center	2370 qualifying patients — 265 (11.2%) developed VTE: 235 DVT only, 19 PE only, 11 both DVT and PE	58 (25%) of all DVT occurred in the first day versus 1 (5%) of all PE; within 2 days of admission, 38% of DVT had occurred versus 26% of PE	For the 19 patients with PE only, all but one of the events were diagnosed within 43 days after admission; risk factors for PE and DVT after injury were different

AIS = Abbreviated Injury Scale; DVT = deep vein thrombosis; IQR = interquartile range; IVC = inferior vena cava; OR = odds ratio; PE = pulmonary embolism; VTE = venous thromboembolism

**Table 2:** Postoperative incidence and time course of pulmonary embolism in surgical patients.

Author (year)	Surgery type and population	PE incidence n/N (%)	Postsurgical time course of PE	Comments
Hope [22] (2007)	General, orthopedic, and other surgical procedures; 1999 to 2004 at a North Carolina medical center	115/111,773 (0.9%)	<40 years of age, mean 2.6 days; 40 to 60 years, mean 11.2 days; >60 years, mean 6.7 days (p = 0.02)	The significantly longer interval to PE diagnosis in patients 40 to 60 years of age possibly due to delays in diagnosis, patient ambulation, and/or asymptomatic PE

Arcelus [23] (2008)	Major orthopedic surgery, cancer surgery, and other procedures; through 2005 in the RIETE registry of patients with symptomatic acute VTE	13,599 registry patients, 1602 postoperative, 787 with PE	19% of PE diagnosed during first 7 days, 48% during first 15 days, 77% during first 30 days	Clinically overt PE appeared significantly earlier than proximal DVT (20 ± 15 days vs 24 ± 16 days, p <0.0001)

Sebastian [24] (2016)	Thoracolumbar surgery; 2005 to 2012 in a national database	202/43,777 (0.5%)	Mean 9.4 ± 7.3 days	For patients undergoing corpectomy, the incidence of PE was 1.7%, mean time to diagnosis 12.4 ± 8.1 days

Jordan [25] (2017)	Nephrectomy; 2006 to 2012 in a national database	65/13,208 (0.5%)	Median 6 days (IQR 3–13); 63.1% of PE occurred prior to discharge	Median length of stay was 8 days for patients who developed PE (IQR 5–11)

Thomas [26] (2018)	Lung cancer resection; 2005 to 2015 in a national database	116/14,308 (0.8%)	Median 11 days (IQR 5–17); 67 (58%) of PE occurred after discharge	Prolonged duration of operation and extended length of stay (both p <0.01) were associated with increased risk for postdischarge VTE

Phillips [27] (2003)	Elective total hip replacement (THR); July 1995 through June 1996 in analysis of Medicare claims data	Primary THR, 0.9% of 58,521; revision THR, 0.8% of 12,956	Approximately 0.3% to 0.4% incidence of PE within the first week after THR, and about 0.7% to 0.8% by the sixth week	26% of PE that developed within 6 months after primary THR occurred during acute inpatient stay, as did 38% of the PE in the revision THR patients

Dahl [28] (2003)	Total hip replacement (THR), knee replacement (TKR), or nailed hip fracture (NHF); 1989 through 1998 in a Scandinavian hospital administering LMWH to all patients	Overall 50/3954 (1.3%); THR, 19/1661 (1.1%); TKR, 3/386 (0.8%); NHF, 28/1907 (1.5%)	Overall mean 27 days (range 1–173); THR mean 35 days (range 5–94); TKR mean 9 days (range 2–17); NHF mean 24 days (range 1–173)	Overall annual PE incidence ranged from 0.4% to 3.4%. PE incidence remained high for at least 1–3 months following major hip surgery, less following TKR.

Bjornara [29] (2006)	Emergency hip fracture surgery or elective total hip replacement (THR) or total knee replacement (TKR); 1989 to 2001 at a Norwegian hospital	Overall, 1.1% of 5607; hip fracture, 32/2420 (1.3%); THR, 28/2512 (1.1%); TKR, 4/675 (0.6%)	Hip fracture, median 17 days (range 1–173); THR, median 34 days (range 3–150); TKR, median 12 days (range 2–150)	The cumulative risk of VTE lasted up to 3 months after hip surgery and for 1 month after TKR; 70% of postsurgery VTE were diagnosed after discharge

SooHoo [30] (2006)	Total knee arthroplasty (TKA); 1991 through 2001 in California linked hospital discharge database	During initial hospitalization, 1.79% of 222,684	PE rate 0.30% during first 30-day period, then declined to 0.06% for second 30-day period; the rate remained in range of 0.03% to 0.05% through the first postoperative year	PE rate stabilized within 30 days after surgery, and proportion of cases was increased by <3% at 60 days; extending follow-up to 90 days had small effect on the proportion of first-year PE cases

Parvizi [31] (2015)	Total joint arthroplasty (TJA), patients receiving postprocedure warfarin; 2000 to 2010 at a university hospital in Philadelphia	283/26,415 (1.07%), within 90 days	Median 2 days (range 1–87 days); 81% within first 3 days, 89% with first week, 94% within 2 weeks	The later PE after revision arthroplasty and in patients without atrial fibrillation possibly due to comorbidity-related delays in postoperative mobility/mobilization

Kim [32] (2016)	Femur fracture in adult patients who underwent CT for PE within 72 hours after admission; January 2010 to December 2014 at a Korean medical center	28/453 (2.2%)	PE detected in 16 (57.1%) of the 28 patients in the first 24 hours after injury, and in 25 (89.3%) in the first 48 hours	PE in femur fracture patients occurred in the immediate period following injury at a relatively higher incidence than commonly appreciated for other types of trauma. However, the outcome of PE in these cases was not fatal.

CT = computed tomography; DVT = deep vein thrombosis; IQR = interquartile range; LMWH = low molecular weight heparin; NHF = nailed hip fracture; PE = pulmonary embolism; THA = total hip arthroplasty; THR = total hip replacement; TKA = total knee arthroplasty; TKR = total knee replacement; VTE = venous thromboembolism

**Table 3:** Selected complications of retrievable IVC filters reported in three systematic reviews and two analyses of the FDA MAUDE database.

Complication	Reports
*Filter fracture* Structural failure of the filter leading to fragmentation	*Reported in specific systematic reviews*
	(i) 0.5% (50/9509 patients) [9]
	(ii) 6 of 14 articles reported fracture rates >10% [7]
	*Percentage of reported MAUDE complications*
	(i) 22.0% (2011) [3]
	(ii) 23.9% (2014) [34]

*Filter migration* Movement >2 cm above or below initial placement	*Reported in specific systematic reviews*
	(i) 1.3% (35/2716 patients) [3]
	(ii) 1.4% (160/11,679) [9]
	(iii) 4 of 14 articles reported migration rates >10% [7]
	*Percentage of reported MAUDE complications*
	(i) 22.0% (2011) [3]
	(ii) 12.1% (2014) [34]

*Filter perforation* Penetration of a filter component >3 mm of vena cava wall	*Reported in specific systematic reviews*
	(i) 5.4% (379/7001 patients) [9]
	(ii) 5 of 14 articles reported perforation rates >20% [7]
	*Percentage of reported MAUDE complications*
	(i) 20% (2011) [3]
	(ii) 15.4% (2014) [34]

*Filter tilt* An angulation >15 degrees from the long axis of the vena cava	*Reported in specific systematic reviews*
	(i) 7.7% (798/10,348) [9]
	(ii) 5 of 14 articles reported tilting rates >10% [7]
	*Percentage of reported MAUDE complications*
	(i) 3.9% (2014) [34]

*Deep vein thrombosis*	*Reported in specific systematic reviews*
	(i) 5.4% (69/1277 patients) [3]
	(ii) 7.1% (362/5092 patients) [9]

*IVC thrombosis/stenosis*	*Reported in specific systematic reviews*
	(i) 2.8% (116/4078 patients), 37 studies [3]
	(ii) 3.9% (345/8788 patients), 103 studies [9]
	*Percentage of reported MAUDE complications*
	(i) 2.3% (2014) [34]

Sources: *Systematic reviews:* Angel et al. (2010) [3], Deso et al. (2016) [7], Jia et al. (2018) [9]; *MAUDE analyses:* Andreoli et al. (2014) [34], Angel et al. (2010) [3]

**Table 4:** Cost and payment data extracted from the 2016 Medicare outpatient hospital standard analytical file for the common procedural terminology code 37193 (IVC filter removal) [57].

Procedure	No anesthesia	With anesthesia	Total
Cases	5547	1140	6687

Median cost (IQR)	$3033.44 ($2385.49 – $3925.33)	$3865.22 ($2909.70 – $5407.56)	$3129.57 ($2457.04 – $4167.18)
Minimum cost	$448.12	$846.91	
Maximum cost	$70,881.80	$37,540.94	

Median payment (IQR)	$1762.54 ($1682.49 – 1897.33)	$1795.42 ($1687.45 – $2080.32)	$1767.49 ($1683.78 – $1921.01)
Minimum payment	$753.52	$809.18	
Maximum payment	$20,797.21	$10,959.56	

IQR: interquartile range
